# Atopic Dermatitis as a Multifactorial Skin Disorder. Can the Analysis of Pathophysiological Targets Represent the Winning Therapeutic Strategy?

**DOI:** 10.3390/ph13110411

**Published:** 2020-11-22

**Authors:** Irene Magnifico, Giulio Petronio Petronio, Noemi Venditti, Marco Alfio Cutuli, Laura Pietrangelo, Franca Vergalito, Katia Mangano, Davide Zella, Roberto Di Marco

**Affiliations:** 1 Department of Health and Medical Sciences “V. Tiberio” Università degli Studi del Molise, 8600 Campobasso, Italy; i.magnifico@studenti.unimol.it (I.M.); n.venditti@studenti.unimol.it (N.V.); m.cutuli@studenti.unimol.it (M.A.C.); laura.pietrangelo@unimol.it (L.P.); roberto.dimarco@unimol.it (R.D.M.); 2 Department of Agricultural, Environmental and Food Sciences (DiAAA), Università degli Studi del Molise, 86100 Campobasso, Italy; franca.vergalito@unimol.it; 3 Department of Biomedical and Biotechnological Sciences, Universitá degli Studi di Catania, 95123 Catania, Italy; kmangano@unict.it; 4 Department of Biochemistry and Molecular Biology, School of Medicine, Institute of Human Virology, University of Maryland, Baltimore, MD 21201, USA; DZella@ihv.umaryland.edu

**Keywords:** atopic dermatitis, skin barrier, cutaneous dysbiosis, *Staphylococcus aureus*, microbial therapy, drug delivery systems

## Abstract

Atopic dermatitis (AD) is a pathological skin condition with complex aetiological mechanisms that are difficult to fully understand. Scientific evidence suggests that of all the causes, the impairment of the skin barrier and cutaneous dysbiosis together with immunological dysfunction can be considered as the two main factors involved in this pathological skin condition. The loss of the skin barrier function is often linked to dysbiosis and immunological dysfunction, with an imbalance in the ratio between the pathogen *Staphylococcus aureus* and/or other microorganisms residing in the skin. The bibliographic research was conducted on PubMed, using the following keywords: ‘atopic dermatitis’, ‘bacterial therapy’, ‘drug delivery system’ and ‘alternative therapy’. The main studies concerning microbial therapy, such as the use of bacteria and/or part thereof with microbiota transplantation, and drug delivery systems to recover skin barrier function have been summarized. The studies examined show great potential in the development of effective therapeutic strategies for AD and AD-like symptoms. Despite this promise, however, future investigative efforts should focus both on the replication of some of these studies on a larger scale, with clinical and demographic characteristics that reflect the general AD population, and on the process of standardisation, in order to produce reliable data.

## 1. Introduction

Atopic dermatitis (AD) is a chronic relapsing inflammatory skin disorder, affecting 7–10% of the adult population and 15–30% of children, and is associated with significant morbidity and decreased quality of life [1]. Although AD can occur at any age, the incidence peaks in infancy with approximately 45% of all cases beginning within the first six months of life, 60% during the first year, and 80–90% by an individual’s fifth birthday [2]. The general term ‘eczema’ was initially used to describe the condition. Subsequently, the correlation between eczema and other atopic disorders led to the coining of the term ‘atopic dermatitis’ in 1933 by Wise and Sulzberger [3]. The AD clinical pattern includes both pruritic and eczematous lesions and the pathophysiology is complex and multifactorial [3,4,5,6]. Current knowledge indicates that the main pathogenetic factors of AD are skin barrier dysfunction and dysbiosis of resident microbiota [7]. To these main factors, immunological dysregulation must be added. Skin barrier dysfunction induces immune dysregulation and immune dysregulation alters skin barrier function. Skin microbial dysbiosis also alters immune responses in AD ([8,9,10]). Therefore, the interaction between barrier dysfunction, microbial dysbiosis and immune dysregulation is at the basis of the worsening of the disease [8]. The skin barrier is localised to the uppermost area of the epidermis, which is the cornified layer (*stratum corneum*) forming by the migration of keratinocytes from the basal to the upper layers. Keratinocytes produce lipids, cyclic adenosine monophosphate (cyclic AMP), cathelicidin and beta-defensins, which form extracellular lipid-enriched layers, kill pathogens and play essential roles in maintaining skin homeostasis [11]. Epidermal barrier proteins, including filaggrin (FLG), keratins, loricrin, involucrin and intercellular proteins, are cross-linked to form an impermeable skin barrier [12]. The alteration in the protein and lipid content of the skin contributes to skin barrier dysfunction. The loss of the function of FLG and other proteins is strongly associated with the development of AD [13]. The overexpression of Th2 and Th22 cytokines altering the protein and lipid content of the skin contributes to skin barrier dysfunction [14]. When developing drug delivery systems (DDSs) for dermatological disorders such as AD, different features of the compromised skin should be considered. In infected, broken or damaged skin where the integrity of the *stratum corneum* is compromised, DDSs improve the efficiency of the formulation [15]. Numerous studies have shown how these systems can aid the delivery of payloads to target sites in dermatological disorder treatment. In particular, the potential for nanocarriers to serve as DDSs for effective AD management has been investigated [15,16].

In addition, an imbalance between *Staphylococcus aureus* (*S. aureus*) and the resident skin microbiota can generate a dysbiosis state that induces an alteration in the immune response and compromises the skin barrier [17]. The skin microbiota plays a role in protecting against infection and inflammation because they guarantee the normal function of the skin barrier. Indeed, viruses, fungi, and bacteria residing on the skin metabolise host proteins and lipids and produce bioactive molecules. These include free fatty acids, cAMP, phenol-soluble modulins (PSMs), microbial cell wall components and antibiotics like bacteriocins that can act on other microbes to inhibit pathogen invasion. All these substances target the host epithelium and stimulate keratinocyte-derived immune mediators such as complement and IL-1, or immune cells in the epidermis and dermis [18,19,20]. For instance, *Staphylococcus epidermidis (S. epidermidis)* suppresses inflammation by inducing the secretion of interleukin-10, an anti-inflammatory cytokine, from antigen-presenting cells [21,22]. In addition, is able to secrete a unique lipoteic acid that suppress both keratinocytes’ inflammatory cytokines and inflammation through a TLR2-dependent mechanism [22,23].

The skin dysbiosis that occurs through an increase in the pathogen *S. aureus* and a variation in the composition and number of skin commensal bacteria also contributes to skin barrier defects and can be a trigger for AD [24]. Indeed, a recent analysis highlighted a prevalence of *S. aureus* on the skin of subjects with AD, with an abundance rate of 70% compared to 39% in the control group [25]. We now have a better understanding of the pathogenetic mechanism of *S. aureus*. This pathogen has numerous virulence factors that contribute to its pathogenesis.

Among these, those most commonly involved in the etiopathogenesis of AD are δ-toxin, phenol-soluble modulins, superantigens, protein A, pro-inflammatory lipoproteins and proteases [26].

In addition to *S. aureus*, skin dysbiosis may occur through an increase in the relative abundance of other species of the genus *Staphylococcus*, such as *S. haemolyticus.* Furthermore, reductions in microorganisms belonging to the genera *Streptococcus* spp., *Propionibacterium* spp., *Acinetobacter* spp., *Corynebacterium* spp. and *Prevotella* spp. have also been observed, which cannot be attributed to an increase in *S. aureus* [27]; on the other hand, *Propionibacterium acnes* was found less frequently on the skin of AD and it was inversely correlated to disease severity [28,29]. After a flare, the species that saw a reduction in their levels then saw an increase in relative abundance [27,29]. An important role is also played by fungal microbiota, which lead to a reduction in the relative abundance of *Malassezia* spp. and an increase in the enrichment of the *M. dermatis* and fungi not belonging to the genus *Malassezia, Aspergillus, Candida* and *Cryptococcus* [29,30,31]. The reconstitution of healthy microbial diversity, presumably by removing *S. aureus* and allowing the skin to repopulate with physiological microbiota, can restore the protective function of the skin and promote the healing process [7,32]. Within the scientific literature, clinical severity has been evaluated using the objective SCORAD index (scoring AD), which was developed by the European Task Force on Atopic Dermatitis (ETFAD) to create a consensus on assessment methods for AD. This system considers both objective signs (severity and extension) and subjective signs (pruritus and loss of sleep). The SCORAD (AD SCORing) allows a unique classification of the disease: mild, moderate or severe. In addition, a complete diagnosis also includes the evaluation of the intensity of the itching [33]. The European guidelines for the management of AD in adults and children are different for the each level of severity: baseline—emollients and bath oils; mild topical glucocorticosteroids; moderate topical tacrolimus or glucocorticosteroids; and severe systemic immunosuppression [34].

In this case, the new treatment options with antibodies (Ab), especially with the Ab Dupilumab, against interleukin-4 receptor revealed great potential without serious side effects [35,36,37,38]

Currently, available drugs are influenced by bioavailability and may give rise to severe adverse events. For example, the use of topical corticosteroids can improve the condition of AD patients, but over-use of corticosteroids during a long bout of sickness can cause some side effects such as hypertension, atrophy and tachyphylaxis result in cumulative toxicity [39]. Although the use of corticosteroids, supported by the use of emollient creams, are widely used in combination to improve symptoms, they do not ensure the complete elimination of AD [40]. The lack of a curative treatment has led to the search for alternative and/or complementary therapies. Microbial therapy and DDSs can help to restore healthy skin microbiota, which have been altered due to skin dysbiosis, and efficiently deliver drugs to skin compromised by AD in order to re-establish the normal function of the skin barrier [41].

This review aims to provide, for the first time, a broad view of AD in light of the newest scientific evidence correlating the two most relevant aspects of this pathology: restoration of healthy skin microbiota and DDSs.

## 2. Results

### 2.1. Microbial Therapy: Restoration of Healthy Skin Microbiota

The use of live/heat-killed or inactivated microorganism, the substances with microorganism-derivatives, and the rebalancing of the physiological skin microbiota through skin bacterial transplant may be considered the therapeutic landscape for AD, since they promote the correct functioning of the skin barrier [7,32,42]. Current scientific evidence shows the role of probiotics in improving the clinical course of AD by restoring skin microbiota homeostasis, maintaining lipid barrier functions and modulating the immune system [43]. In addition, some bacterial compounds such as cell wall fragments and their metabolites demonstrate greater stability than viable cells when kept at room temperature, making them more suitable for the formulation of topical preparations. For example, microbe free cultures are still able to exert antimicrobial and immunomodulatory activity in the same way as vital forms [44]. Lastly, studies on the effects of bacterial skin transplant (SBT), an intriguing treatment for the restoration of a healthy skin microbiome in AD patients, have yielded promising results in human and animal models [45]. Together, these approaches have low costs, few side effects, a more relaxed therapy (no daily application necessary) and a more lasting effect.

#### 2.1.1. Live Microorganisms

The use of living microorganisms as food supplements or in medical practices for the treatment of bacterial vaginosis, vaginitis, childhood colic, obesity, type 2 diabetes and pharingotonsillitis is already well known [46,47]. Clinical and experimental research extensively documents the capacity for probiotics to go beyond positively influencing the intestinal functions, and to exert their benefits at the skin level thanks to their peculiar properties [43]. The topical administration of probiotics can increase skin ceramides, improve erythema, scaling and pruritus, and decrease the concentration of the pathogenic *S. aureus* [48].

There have been several studies into the use of live microorganisms for the treatment of AD, using both human and animal models. Seven of these studies are reviewed: three employed animal models; four involved clinical trials, of which three involved children and one adults (see Table S1A in the Supplementary Electronic Material for details).

Firstly, an in vivo study using Sprague-Dawley rats and ddY mice, and the oral administration of *Lactobacillus plantarum*. It has been proven that food supplementation of β-1,3/1,6-glucan and/or *L. plantarum LM1004* can reduce vasodilation, itching, oedema and regulates the immune response [49].

In a double-blind clinical trial on 50 children with moderate AD, the oral administration of a mixture of the probiotics *Bifidobacterium lactis, Bifidobacterium longum* and *Lactobacillus casei* was effective in reducing SCORAD index scores and reducing the use of topical steroids to treat flares when compared to the control arm. These findings suggest that such a mixture of probiotics can be used for the treatment of AD [50].

An in vivo study on SKH-1 hairless mice aimed to test a probiotic mixture of five bacterial strains, *Bifidobacterium longum*, *Lactobacillus helveticus*, *Lactococcus lactis*, *Streptococcus thermophilus* and *Lactobacillus rhamnosus*, in preserving skin integrity and homeostasis. It has been observed that daily oral treatment with the probiotic mixture, through modulation of the immune response, has significantly limited chronic skin inflammation, demonstrating its use in pathological dermatological conditions such as AD and psoriasis [51].

The oral administration of *Weissella cibaria* WIKIM28 in a mouse model of AD induced in BALB/c mice has shown that this bacterial strain can be a good candidate as a probiotic for AD prevention and improvement. Thus, the intake of this live microorganism improved AD-like skin lesions and exhibited excellent immunomodulatory activity [52].

A randomised, double-blind study carried out on 220 children affected by moderate/severe AD, showed that the oral administration of *Lactobacillus paracasei* and *Lactobacillus fermentum*, for 3 weeks led to decreased IgE, TNF-α, urine eosinophilic protein X and SCORAD scores. Thus indicating that supplementation of a probiotic mixture of *L. plantarum* and *L. fermentum* is associated with clinical improvement of AD [53].

Another trial on 43 children tested *Lactobacillus salivarius*, which, when orally administered, showed a significant improvement in clinical parameters, SCORAD scores and itch values [54].

In a prospective controlled pilot trial on 25 adults, the oral administration of the probiotic strain *L. salivarius* LS01 in association with *Streptococcus thermophilus*, significantly improved both SCORAD scores and the *S. aureus* count. Moreover, the combination of *S. thermophilus* ST10 with *L. salivarius* LS01 improved the overall effectiveness of the formulation by reducing the recovery time [55].

#### 2.1.2. Heat-Killed or Inactivated Microorganisms.

The growing interest in the biological effects of heat-killed or inactivated microorganisms is already well documented. In particular, the use of heat-treated probiotic bacteria (lactic and bifidobacteria), together with their cell-free supernatants or selected purified cellular components in immunomodulation and maintaining the integrity of the intestinal barrier against enteropathogens is well known to the scientific community. Only recently, numerous scientific studies have investigated the role of these non-viable microorganisms in the management of dermatological diseases [56]. There are several studies that have investigated the potential of heat-killed or inactivated microorganisms for the treatment of AD, which have used both human and animal models. The findings of seven of these studies are reported herein. One employed animal models, five involved clinical trials, of which two were in children, and finally one was conducted within an in vitro reconstructed human epidermis (RHE) (see Table S1B in the Supplementary Electronic Material for details).

Topical application of a formulation containing heat-treated *Lactobacillus johnsonii* NCC 533 (HT La1) was able to modulate endogenous antimicrobial peptides (AMP) expression and to inhibit the binding of *S. aureus* in an in vitro reconstructed human epidermis model (RHE). These results highlight the role of innate skin immunity in reducing *S. aureus* colonization in atopic skin [57].

An open-label clinical study in AD patients showed that the application of a lotion containing a heat-treated *Lactobacillus johnsonii* NCC 533 (HT La1) led to a decrease in the SCORAD score. This clinical improvement was associated with a reduction in the *S. aureus* viable count. In addition, the authors were able to establish a directly proportional correlation between the *S. aureus* skin concentrations and the lotion response [58].

In a double-blind clinical trial conducted on 60 patients suffering from moderate AD, topical application of an emollient containing biomass from the non-pathogenic bacteria *Vitreoscilla filiformis* lysate one month after the end of the treatment ameliorated the evolution of the average SCORAD score, which was significantly lower than that of the control patients treated with a generic emollient.

During one month of treatment, the level of *Staphylococcus* spp. decreased in treated subjects with the formulation enriched by *V. filiformis* biomass, demonstrating the normalization of the skin microbiota and the significant reduction in the number and severity of flare-ups compared to another formulation without bacterial biomass [59].

In a clinical trial on 179 children, oral administration of the bacterial lysate OM-85 of 21 strains from eight common respiratory pathogenic microorganisms (i.e., *Haemophilus influenzae, Streptococcus pneumoniae, Klebsiella ozaenae and pneumoniae, S. aureus, Streptococcus viridans* and *pyogenes* and *Neisseria catarrhalis*) showed an adjuvant therapeutic effect which led to significantly fewer new flares and delayed their onset. Indeed, these results showed an adjuvant therapeutic effect of a well-standardised bacterial lysate OM-85 on established AD [60].

An in vivo study on NC/Nga mice demonstrated that the oral administration of *Lactobacillus plantarum* lysates was able to restore the skin homeostasis of the treated animals. Indeed, after two months of treatment, there was a reduction in the formation of the horny layer and a decrease in skin thickening compared to untreated mice [61].

Kim et al. stressed the importance of clinical research in the study of AD. In their study, the authors tested *L. plantarum* K8 lysates formulation, both with in vitro/in vivo experiments, and in a clinical trial with the healthy volunteer. Preliminary data obtained in vitro with HaCaT cells and after 2 months of in vivo treatment with on DNCB-treated SKH-1 hairless mice demonstrated an attenuation of the stratum corneum formation and epidermal thickening of AD mice skin. These data were supported by the clinical study, where an improvement in the barrier function of the epidermis was observed in subjects who ate candies containing L. plantarum K8 lysate [62].

In a clinical trial, 606 infants at risk of atopy were treated with an oral application of bacterial lysate containing heat-killed *Escherichia coli* and *Enterococcus faecalis*. The results showed a reduced possibility of developing AD, suggesting that bacterial lysates prevent the development of this skin condition in children [63].

#### 2.1.3. Microorganism-Derived Substances

The capacity of microorganism-derived compounds to inhibit allergic inflammation make them candidates for novel therapies for allergic diseases [64]. Among these compounds are bacteriocins, proteins and enzymes [65]. Several studies have highlighted the beneficial role of skin commensals due to the production of bacteriocins. Indeed, many members of the cutaneous microbiome can metabolise glycerol into antimicrobial compounds, such as bacteriocin, that inhibit *S. aureus* growth. Skin commensal coagulase-negative staphylococci (CoNS) are the primary producers, but there are also other microorganisms able to produce these compounds [66,67]. There are several studies, both in human and animal models. In this section, seven studies concerning the use of microorganism-derived substances for AD treatment are presented. Of these, four employed animal models, two were conducted in vitro, and one were conducted both in vitro and in vivo (see Table S1C in the Supplementary Electronic Material for details).

An in vitro study showed that cytoplasmic bacteriocins isolated from *S. epidermidis* selectively exhibited antimicrobial activity against *S. aureus* and methicillin-resistant *S. aureus* (MRSA). These findings suggest that these cytoplasmic bacteriocin compounds could potentially inhibit the growth of *S. aureus* and be used as a topical AD treatment [68].

In an in vivo model of AD on BALB/cAJcl mice, the oral administration of an exopolysaccharide (EPS) produced by *Lactobacillus paracasei* reduced ear swelling, produced a repression of ear interleukin-4 (T helper (Th) 2 cytokine) mRNA and decreased serum immunoglobulin E levels. These results suggest that *Lactobacillus paracasei*-derived EPS inhibits the catalytic activity of hyaluronidase promoting inflammatory reactions and is useful for improving type I and type IV allergies, including AD [69].

The commensal yeast *Malassezia globosa*, secretes a protease called ‘*Malassezia globosa* secreted Aspartyl Protease 1 (MgSAP1)’, which, in vitro, can disrupt *S. aureus* biofilms by hydrolysing protein A. This study defined a role for the skin fungus Malassezia in inter-kingdom interactions and suggested that this fungus enzyme may be beneficial for skin health [70].

In a mouse model the topical application of p40, a particulate fraction from *Corynebacterium granulosum*, used in a formula with hyaluronic acid produced a significant reduction in ear thickness, weight, oedema, and leukocyte recruitment. These results suggest that p40-conjugated with hyaluronic acid may constitute an outstanding innovative dermatitis treatment [71].

In addition, other bacteria not belonging to the skin microbiota are able to produce antibiotics with properties useful for treating AD. An example would be the topical application of josamycin, a macrolide antibiotic derived from *Streptomyces narbonensis* subsp. *josamyceticus* which was applied to NC/Nga mice. In this case, the topical application of this antibiotic reduced the expression of proinflammatory cytokines demonstrating antimicrobial activity against *S. aureus* present on the skin of AD mice [72].

Another molecule with antibacterial activity, produced by *S. lugdunensis*, lugdunine, was tested in an in vivo experiment with shaved black-6 (C57BL/6) mice and it was able to reduce or completely eradicate *S. aureus* viable count both on the surface and in the deeper layers of the skin. The isolation and study of other lugdunin- or lugdunin-like molecules isolated from s commensal bacteria could represent a new therapeutic approach in the prevention and management of staphylococcal infections [73].

Similarly, an AD-like in vivo NC/Nga mice model demonstrated that the protein P14, isolated from *Lactobacillus casei*, can be used as an active immunomodulatory agent for treating patients with AD [74].

#### 2.1.4. Skin Bacterial Transplantation

Although there are still few studies on the transplantation of skin bacteria (SBT), this particular type of bacteriotherapy that involves transplanting several skin microbiota from one individual to another has already provided promising results in both human clinical trials and in animal models [45]. Indeed this intriguing therapeutic potential has earned it the definition of the “future of eczema therapy” [75]. Herein, three human studies are reported focusing on skin microbiota transplantation for the treatment AD. Of these studies, one involved a clinical trial conducted on healthy volunteers to develop the technique for transferring the entire skin microbiota, another was carried out on adults, and the last one involved both adults and paediatric patients (see Table S1D in the Supplementary Electronic Material for details).

In a recent prospective pilot study, researchers attempted to perform a complete skin microbiota transplant that shifted the entire bacterial skin community of healthy volunteers from the forearm to the back in a unidirectional manner. Evidence of the transfer of a partial DNA signature was seen by comparing the bacterial species present in the arm with the mixed communities (‘transplantation’) that were absent in the back. This technique aimed to move viable skin organisms from one site to another and is worthy of further investigation [76].

The successful transplantation of *Roseomonas mucosa* was conducted in an open-label phase I/II safety and activity trial with adults and pediatric patients. The results demonstrated a significant decrease in disease severity, a reduction in steroid administration, and a viable *S. aureus* count [77]. All these finds were supported by a previous study in mice conducted by the same authors [20].

Najatsuji et al. conducted a clinical study by autologous CoNS transplantation isolated from AD patients *S. aureus* culture positive. After isolation, CoNS strains (*S. epidermidis* and *S. hominis*) were formulated in a cream base vehicle and applied to the forearm of the same subjects for 24 h. The results showed a significant decrease in *S. aureus* colonization at the microbial transplant site compared to the contralateral forearm treated with the bacteria-free vehicle alone. These observations were also confirmed by in vivo experiments on the back of C57BL6 mice. These findings show, once again, the role of commensal skin bacteria in protecting against colonisation by pathogens and how dysbiosis of the skin microbiome can contribute to the onset of the disease [19].

### 2.2. Drug Delivery Systems

It is often preferable to use non-invasive delivery to provide relief for AD [78]. Topical treatment is preferential to parenteral or oral administration because of better compliance and the reduction in drug concentrations and side effects [79]. Topically, DDSs deliver therapeutic agents or natural active compounds directly to the target site to maximise the benefits and minimise the risks associated with drugs. In this regard, in the last two decades, an interest in nano-based DDSs has developed. The latter have already been applied in the treatment of various diseases ranging from cancer to Alzheimer’s [80].

The most common nano-based DDS carriers addressed in this manuscript, include polymeric nanoparticles (NPs), solid lipid nanoparticles (SLNs), lLiposomes, ethosomes, and elastic vesicles due to their small size (range from 1 to 1000 nm). They can penetrate through the *stratum corneum* and accumulate in the target site, improving the delivery of transported bioactive compounds and favouring higher drug retention, demonstrated by drug diffusion and permeation study profiles [79,80,81,82]. Although the dimensions are variable, desired therapeutic benefits, avoidance of off-target effects, and optimal localised delivery of drugs are achieved using nanocarriers <200 nm in size. Nanocarrier-mediated interventions have been well-reported for topical and transdermal applications [83]. Together, these approaches offer novel solutions, allowing: (i) the management of severe forms of AD, especially those not responsive to steroid therapy; (ii) improved performance of pharmacokinetic parameters such as permeation and controlled release; (iii) significant improvements in the patient’s state of health; iv) a reduction in the dosage of the active ingredient with a consequent reduction in toxicity and an improved safety profile [84,85].

#### 2.2.1. Nanoparticles

Nanoparticles (NP) are a broad class of DSS in the order of 100 nanometres with optimal rheological properties, antimicrobial effects and the ability to restore skin conditions [16,86]. For instance, NPs loaded with a lipid drug and/or made by lipophilic compounds (i.e., lipid NPs) ensure skin hydration and the occlusion effect in a size-dependent manner and can form a thin film on the skin surface, which allows for rehydration [87]. The complete biodegradation of lipid NPs and their biocompatible chemical features have secured them the title of nano-safe carriers [84]. Twelve studies concerning the use of NPs in AD treatment were identified for review. Only in vivo studies using animals were selected. Of the seventeen studies, one employed only in vivo animal models, four were conducted in vitro and ex vivo, six were conducted in vitro and in vivo, and one was conducted in vitro, ex vivo and in vivo. In vitro tests provided a characterisation and evaluation of the formulation (see Table S2A in the Supplementary Electronic Material for details).

An in vitro and ex vivo drug test performed using a jacketed Franz diffusion cell showed that nanoencapsulation of betamethasone valerate (BMV) into the chitosan nanoparticles (CS-NPs) displayed a Fickian diffusion type mechanism of release in the simulated skin surface. Drug permeation efficiency and the amount of BMV retained in the epidermis and the dermis was higher when compared to BMV solution alone. These results suggest that this formulation of betamethasone improved the therapeutic efficacy of the treatment of AD [88].

Tacrolimus-loaded thermosensitive solid lipid nanoparticles (TCR-SLN) in the dorsal skin of Sprague Dawley rats penetrated to a deeper layer than the control formula. The penetration test in vivo of the skin of white rabbits demonstrated that TCR-SLNs delivered more drug into deeper skin layers than the control, suggesting that thermosensitive SLNs could be employed for the delivery of difficult-to-permeate, poorly water-soluble drugs into deep skin layers [89].

In an in vitro test with a Franz static diffusion cell system and ex vivo on skin from Wistar albino rats, the application of ‘hyaluronic acid-modified betamethasone encapsulated polymeric nanoparticles’ (HA-BMV-CS-NPs) revealed that drug permeation efficiency of betamethasone was higher in the case of BMV-CS-NPs and that there was a greater amount of drug retained in the epidermis and the dermis. This complex could be a promising nano delivery system for efficient dermal targeting of BMV and improved anti-AD efficacy [90].

In a clinical trial that enrolled healthy volunteers treated with hydrocortisone hydroxytyrosol anti-oxidant-loaded chitosan nanoparticles (HA-HT-CSNPs) to evaluate systemic toxicity, the results of blood haematology, blood biochemistry, and adrenal cortico-thyroid hormone levels were not significant. This indicated non-systemic toxicity and supports the view that this formula could be used for AD treatment [91].

In vitro and in vivo permeation studies on Sprague Dawley rats with tacrolimus nanoparticles based on chitosan and combined with nicotinamide (FK506-NIC-CS-NPs), demonstrated that these nanoparticles significantly enhance tacrolimus permeation through and into the skin, and deposited more tacrolimus into the skin. Moreover, this system enhances the permeability of tacrolimus and plays an adjuvant role in anti-AD, reducing the dose of tacrolimus in treating AD, and is, therefore, a promising nanoscale system of tacrolimus for the effective treatment of AD [92].

Betamethasone Valerate incorporates in a lipidic carrier revealed an enhancement of the Betamethasone Valerate ratio in comparison with the control group and had an anti-inflammatory effect. The outcome of complete characterisation suggests that the developed formulation is efficient in a single daily dosage in the therapy of AD [93].

An in vitro/ex vivo test on NC/Nga mice skin demonstrated the anti-AD efficacy of tacrolimus-hyaluronic acid-charged nanoparticles. According to the author’s findings, this formulation can be used as a promising therapeutic approach for patients who cannot be treated with steroid therapy, such as children and adults with steroid intolerance [94].

In an in vivo test with SKH-1 mice, the topical application of dendritic nano-multi-shell dendritic nanocarriers was evaluated as a deposit formulation for anti-inflammatory drugs in the skin. Both in vitro release and toxicological studies have confirmed the biocompatibility of the formulation, providing evidence of prolonged release of the active substance especially for anti-inflammatory drugs like those used in AD. Furthermore, no evidence of local or systemic toxic/adverse effects was observed [95].

An in vivo test on Wistar albino rats evaluated the penetration into the deep skin layers of cationic polymeric chitosan nanoparticles loaded with anti-inflammatory (hydrocortisone) and antimicrobial (hydroxytyrosol,) anti-inflammatory agents compared to a similar commercial formulation. The results proved a better performance in the local release of the active ingredients without involving the underlying tissues. In addition, no toxicity was found compared to the commercial formulation, providing substantial safety benefits [96].

In an in vivo test with NC/Nga mice, transcutaneous co-delivery based on nanocarrier hydrocortisone and hydroxytyrosol was studied as a possible therapy for the management of the immunological and histological issues of AD. The results of immunological and histological experiments conducted on the sera and biopsies of the tested mice confirmed this hypothesis [97].

Furthermore, a Silver-nano lipid complex incorporated into an o/w cream and a lotion showed a high adhesivity to the skin and bacterial surfaces, leading to a locally high concentration of silver ion killing bacteria, restoring the distorted skin barrier, and being much more useful than silver alone. Data were generated either by in vitro tests determining the colony-forming unit (CFU) count over time of *S. aureus* ATCC25923, or in vivo on BALB/c mice. This formula makes the drug more effective in terms of enhanced penetration and exploits the skin normalisation ability of the skincare sNLC formulation [16].

Another in vivo study in NC/Nga mice aimed to assess whether the transcutaneous administration of hydrocortisone nanoparticle could be considered a valid therapeutic approach in the management of dermatitis suggested a substantial reduction in inflammatory cascade mediators, accompanied by positive histological results on fibroblast infiltration and elastic fiber fragmentation, demonstrating how these formulations can promote and maintain the integrity of connective tissues especially in an injured skin like AD [98].

#### 2.2.2. Liposomes, Ethosomes, and Elastic Vesicles

Liposomes and ethosomes can be defined as vesicular DDSs. Liposomes are spherical vesicles with particle sizes ranging from 30 nm to several micrometres consisting of single or multiple concentric lipid bilayers encapsulating an aqueous compartment. These formulations have been successfully applied for the management of AD due to their moisturising effect on the *stratum corneum* and their ability to act as bioactive compound carriers [85]. Rigid liposomes remain confined to the *stratum corneum*, resulting in the formation of a drug reservoir in the upper skin layers, and do not allow percutaneous absorption. More recently, efforts have been made to investigate vesicular lipid systems capable of facilitating drug penetration to the underlying skin layers, allowing transdermal absorption [99].

In contrast, ethosomes are made mainly of phospholipids with a high concentration of ethanol (20–50%) and water. Due to this composition, they have demonstrated remarkably high deformability features [100]. Moreover, ethosomes guarantee a more efficient transfer of the active principle through the skin (epidermis and dermis) than liposomes [15].

Finally, a further advance in the field of DDS is represented by the elastic vesicles used as a new topical and transdermal delivery system. Although the manufacturing method of these vehicles is very similar to that of liposomes, the presence of an ‘activating’ agent in the phospholipid bilayer gives it a high degree of elasticity. It has been demonstrated that the topical administration of elastic vesicles does not occlude the skin and easily permeates through the *stratum corneum* lipid lamellar regions due to skin hydration or by osmotic force. Furthermore, this DDS can be loaded with a wide range of small molecules, peptides and proteins [101].

Six applications of liposomes, ethosomes, and elastic vesicles in AD treatment are herein reported. Of them, one was conducted using only in vitro methods, one enrolled patients with AD, one were conducted by in vitro and ex vivo studies, one by in vitro and in vivo and in the last two an in vitro, ex vivo and in vivo methodology was adopted. In vitro tests have provided a characterisation and evaluation of the formulation (see Table S2B in the Supplementary Electronic Material for details).

In an in vitro test with a static Franz diffusion cell setup on the heat-separated human epidermis, the use of ultra-flexible lipid vesicles effectively delivered cyclosporin A into the skin. This study introduces a promising approach to the topical treatment of skin pathologies with an immune component [102].

In an in vitro test with a dialysis membrane and ex vivo with Wistar rat skin, the application of cyclo-ethosomes with fluocinolone acetonide (FA) showed maximum permeability as compared with an optimised reference ethosomal gel and control gel. These results suggest that β-cyclo-ethosomes could be a promising carrier for improvised penetration of fluocinolone acetonide via topical gel [103].

In an open-label pilot study of 20 patients with AD, the application of liposomal polyvinylpyrrolidone-iodine hydrogel showed that this strategy was well tolerated and led to an improvement in pain, quality of life, eczema area and severity. This formula has potential utility as an effective treatment for inflammatory skin conditions associated with bacterial colonisation [104].

An in vitro test with a dialysis membrane and ex vivo with Wistar rat skin revealed that nano ethosomal glycolic vesicles of triamcinolone acetonide have excellent permeation. Besides the histological analysis, the study confirmed the non-irritant potential. These results suggest that nano-ethosomal glycolic vesicles can be active non-irritant carriers for the improvised penetration of triamcinolone acetonide for potential topical therapeutics [105].

The pharmaco-dynamic evaluation of the ethosome-based topical delivery system of the antihistaminic drug cetirizine (measured by in vivo and ex vivo tests on BALB/c mice) showed a reduction in the scratching score, the erythema score, skin hyperplasia and the dermal eosinophil count. The data suggest that this formula could be an effective carrier for the dermal delivery of the antihistaminic drug, cetirizine, for the treatment of AD [106].

An in vivo and ex vivo tests on BALB/c mice, a topical formulation of levocetirizine based on flexible vesicles (FVs) showed a reduction in the scratching score and the erythema score in addition to the dermal eosinophil count [107].

## 3. Discussion

AD is a pathological skin condition that is becoming increasingly common in clinical dermatological practice. The pathogenesis is exacerbated by its complex aetiological mechanisms that are not yet fully understood, providing many opportunities for misinterpretation [108]. Among the different hypotheses, numerous studies have demonstrated that dysbiosis and skin barrier dysfunction contribute to the pathobiology of AD [109]. Immune dysregulation is another factor involved in the pathogenesis of AD and is closely related to the previous ones. Indeed skin colonisation of Staphylococcus aureus damages the skin barrier and induces inflammatory responses, on the other hand, local Th2 immune responses diminish barrier function, promoting bacterial dysbiosis [9].

Although it is common to associate skin dysbiosis with an increase in *S. aureus* abundance, more recent studies are converging on the opinion that AD skin microbiota is characterised by low bacterial diversity. The relative abundance of both *S. aureus* and *S. epidermidis* are elevated and the presence of *Propionibacterium* spp. is reduced, along with other genera (*Streptococcus*, *Acinetobacter*, *Corynebacterium* and *Prevotella*). Moreover, the absence of early colonisation with commensal staphylococci might precede AD presentation [31]. Skin dysbiosis contributes to skin barrier defects [12]. The latter promote easy penetration of numerous insults relevant to the development of the disease i.e., pathogens, toxins, allergens, irritants and pollutants. Accordingly, all the treatments (pharmacological and adjuvants) aim to minimise the number of exacerbations, the so-called ‘flares’, and reduce their duration and intensity [110]. To date, there is not a resolutive therapy that can take into account the complex pathogenic interplay between a patient’s susceptible genes, their skin barrier abnormalities and their immune dysregulation [15].

The majority of AD patients are paediatric and when moderate-to-severe symptoms occur, current therapies have proven to be of limited efficacy and have several side effects [111,112,113]. For all these reasons, there has been a surge of interest from clinicians and the lay public in exploring targeted bacteriotherapy to treat this pathological skin condition [76]. Microbic therapies with microorganisms that are commensal of the healthy skin microbiota, or probiotics in conjunction with transplantation, could represent a new diagnostic and therapeutic target for AD [114,115,116]. Several studies have demonstrated that probiotic use has led to increased skin ceramides and has improved erythema, scaling and pruritus, suggesting that probiotics may be useful for the treatment of AD, especially for moderate to severe AD in children and adults [48,51,53,116]. Furthermore, specific probiotic strains have shown active immunomodulatory properties [59,117].

Restoring the skin microbiota homeostasis could also represent a new era in AD treatment [118]. The reconstitution of healthy microbial diversity can boost the right immune response and normal barrier function [7,32,119,120]. Similarly, other studies have demonstrated that commensal microorganisms can reduce *S. aureus* by bacteriocin production or competition mechanisms, improving AD symptoms. In this context, the development of antibiotic resistance by the *S. aureus* methicillin-resistant (MRSA) strain has considerable importance, not only from the point of view of infectious disease but also as it can influence the course of the disease. Bacteriocins from CoNS also exhibit antimicrobial activity against MRSA [72,121]. The clinical promise of transplanting commensal skin organisms from healthy individuals onto diseased skin, together with faecal microbiota transplantation to selectively target pathogenic *S. aureus,* thus modifying the diseased skin microbiome to attenuate the course of the disease, have been investigated, with promising results [16,76].

Furthermore, the therapeutic potential of DDSs based on nano-products has provided a new avenue for the prevention and treatment of inflammation and sequelae of skin diseases. Several studies have shown the effectiveness of nanoparticles, liposomes, ethosomes and vesicles in AD. This was particularly valid in recalcitrant form treatments, due to their unique characteristics, such as the improvement in pharmacokinetic parameters (targeted transdermal release of the active ingredient, permeation, retention, and diffusion) and physicochemical properties. These advances in pharmaceutical technology have led to improvements in both clinical symptoms and immune responses, along with better inhibition of inflammatory cascades mediators that positively impact patients’ quality of life, with fewer adverse events reported and increased patient compliance [85,110,122,123].

## 4. Materials and Methods

The interest of the scientific community in research into novel targets for the development of effective therapeutic strategies in AD management has dramatically increased. For this reason, the bibliographic research for scientific papers specialised in the field of interest was conducted from 2014 to March 2020 on PubMed (the MEDLINE database), using the following keywords: ‘atopic dermatitis’, ‘bacterial therapy’, ‘drug delivery system’ and ‘alternative therapy’ alone and/or in combination. As a preliminary result, more than 300 documents were found. Of these, 24 papers on microbial therapy and 15 on nano-based DDSs were selected for review due to their relevance.

## 5. Conclusions

All the studies reviewed show enormous potential for AD treatment, so we can state that research into novel targets is key to the development of effective therapeutic strategies. Nevertheless, some limitations still need to be overcome. An aspect of primary importance in the advancement of scientific and technological innovation is the possibility of marketing the new formulations. To this end, there are different international regulations regarding bacterial formulations for medical use. The European Medical Device Directive (MD) (DDM 93/42) and subsequent amendments include MDs containing live microorganisms (especially those containing probiotics) for the management of AD [124]. On the other hand, the US Food and Drug Administration (FDA) has not approved any oral or topical microbial-based formulations for the treatment of dermatological condition [125].

Although the potential of bacteriotherapy for the treatment of AD seems to be clear, further studies will need to be conducted with the goals of recruiting more patients with different clinical characteristics and standardising the process to produce reliable data. Put differently, even if the topically used DDSs offer promising opportunities in dermal delivery, many questions arise, which remain to be explored and addressed, concerning, for example, their toxicological characteristics and the long-term safety of these technologies.

In vivo and in vitro assays are useful to identify the toxicity of dds because they help to establish the dose–response relationship [126]

However despite in vitro tests are useful for bypassing cell interactions that exist in vivo, in vivo toxicity testing is needed due to the difference between in vitro dosimetry and real topical exposure and additional innovative research is needed to address the cost-effectiveness and long-term safety of these nanoparticles [127].

## Supplementary Materials

The following are available online at https://www.mdpi.com/1424-8247/13/11/411/s1, Table S1: Restoration of healthy skin microbiota, Table S2: Drug Delivery System (DDS) for AD treatment.
