# Supplementary material for: Atopic Dermatitis as a Multifactorial Skin Disorder. Can the Analysis of Pathophysiological Targets Represent the Winning Therapeutic Strategy?
Source: Pharmaceuticals (Basel). 2020 Nov 22;13(11):411. doi: 10.3390/ph13110411 (PMC7700401; doi:10.3390/ph13110411)
Supplement: Supplementary file 1 [file pharmaceuticals-13-00411-s001.pdf]

Table S1. Restoration of healthy skin microbiota. A) Live microorganism

	Microorganisms (manipulation methods and/or derivatives)	*In vivo*, *in vitro* or *ex vivo* methodology used	AD Stage or Induction method / Route of administration	Results	Conclusion	Ref.	Year
LIVE MICROORGANISMS	*L.plantarum* LM1004	SD rats and ddY mice	Histamine-induced vasodilation AD and Contact dermatitis induced by dinitrophenyl- derivatised ovalbumin/ Oral	• Reduced vasodilation, pruritus, oedema, and serum histamine; • decreased expression levels of Th2 and Th17 cell transcription factors • enhanced transcription of immunomodulation factors (Th1 and Treg cells, galactin-9 and filaggrin).	The potential for AD treatment was demonstrated by mechanisms that might involve the modulation of host immune systems and gut microbiota.	[129]	2019
	B. lactis CECT 8145 *B. longum* CECT 7347 *L.casei* CECT 9104	Clinical trial (Children)	Moderate/ Oral	• Improvement in SCORAD; • reduction in the use of topical steroids.	Reduction of SCORAD index and use of topical steroids in patients with moderate AD.	[50]	2018
	B. longum LA 101 *L.helveticus* LA 102 *L. lactis* LA 103, *S. thermophilus* LA104 *L. rhamnosus* LA 801	Hairless SKH-1 mice	Chronic skin inflammation induced by 2-O-tetradecanoylphorbol-13-acetate / Oral	• Induced chronic skin inflammation limited; • downregulation of pro-inflammatory cytokines (IL-1β, IL-6, TNF-α, IL- 17 and IL-22); • up-regulated levels of the anti-inflammatory cytokines, IL-10, and IL-4.	Help in preserving skin integrity and homeostasis.	[51]	2018

**LIVE MICROORGANISMS**	*W. cibaria* WIKIM28	BALB/c mice	AD-like lesions induced by 2,4-dinitrochlorobenzene / Oral	• Reduction in skin lesions, epidermal thickening, and serum immunoglobulin E levels;<br>• type 2 T cells cytokines decreased;<br>• the proportion of CD4$^+$CD25$^+$Foxp3$^+$regulatory T cells in mesenteric lymph nodes and IL-10 levels in polyclonal stimulated MLN cells were both increased.	Amelioration of AD-like symptoms by suppressing allergic Th2 responses and induction of Treg responses.	[52]	2017
	L. paracasei<br>*L. fermentum*	Clinical trials (children)	Moderate to severe/ Oral	• Improvement in SCORAD	Significant clinical improvement.	[53]	2015
	L. salivarius LS01	Clinical trials (children)	Moderate to severe/ Oral	• Improvement in SCORAD	Significant clinical improvement.	[54]	2014
	L. salivarius LS01<br>*S. thermophiles* ST10	Pilot trial (Adults)	Moderate to severe/ Oral	• Improvement in SCORAD<br>• Slight decrease in faecal *S. aureus* count	The potential role of the two bacterial strain for AD treatment was demonstrated, moreover the addition of tara gum improve the overall efficacy of the probiotic strain.	[55]	2014

Table S1 Restoration of healthy skin microbiota (Continued. B) Heat-killed or inactivated microorganisms

	L. johnsonii NCC 533 (heat-killed)	*In vitro* reconstructed human epidermis (RHE)	*S. aureus in vitro* adhesion to skin and boost cutaneous innate immunity/ Topical	• Reduction of the binding of radiolabelled *S. aureus;*<br>• antimicrobial peptide expression induction.	An enhanced cutaneous innate immunity and reduced *S. aureus* colonisation was demonstrated.	[57]	2018
**HEAT-KILLED OR INACTIVATED MICROORGANISMSI**	*L. johnsonii* NCC 533 (heat-killed)	Clinical trial (adult)	Mild-to-moderate/ Topical	• Reduction in *S. aureus* count;<br>• improvement in SCORAD.	The findings support further development of topical treatments containing heat-treated non-replicating beneficial bacteria for AD treatment.	[58]	2017
	V. filiformis (biomass)	Clinical trial (aged from 6 months to 63 years)	Moderate form of AD / Topical	• Treatment with a lipophilic cream containing a biomass of *V. filiformis*<br>○ Improvement in SCORAD<br>○ Increased level of *Xanthomonas* genus<br>• Stable level of *Staphylococcus* genus.	The ability to normalise skin microbiota and a reduction in the number and severity of flare-ups was demonstrated.	[59]	2017
	H. influenzae, S.pneumoniae, K. ozaenae K. pneumoniae, S. aureus, S.viridans S. pyrogenes, N. catarrhalis (OM-85 bacterial lysate)	Clinical trial (Children)	mild to severe / Oral	• Occurrence of new flares was reduced and/or delayed;<br>• no major side effect observed together;<br>• good tolerability.	Clinical efficacy and long-term tolerability of an oral bacterial extract, as adjuvant therapy in children with established AD was demonstrated.	[60]	2017

**HEAT-KILLED OR INACTIVATED MICROORGANISMSI**	*L. plantarum* KCTC 10887BP (lysate)	NC/Nga mice back skin	Skin sensitisation/ Oral	• Horny layer formation attenuation;<br>• reduction of epidermal thickening;<br>• increase in epidermal permeability barrier function;<br>• reduced spontaneous scratching behaviour.	Host homeostasis improvement and utilisation for the clinical treatment of inflammatory diseases.	[61]	2015
	L. plantarum K8 (lysate)	SKH-1 hairless female mice and Clinical trial (Adults)	Mice skin sensitisation and Healthy Volunteers / Oral	• Mouse model:<br>○ attenuation in horny layer formation<br>○ decreased epidermal thickening<br>○ increase in epidermal permeability<br>○ reduced damage to barrier function<br>• Clinical study:<br>○ improvement in barrier repair and function	The alleviation of AD lesions in the mouse model indicates that *L. plantarum* K8 lysates have a moisturising effect. Clinical trial results showed increased hydration, less water loss, and decreased horny layers on the face and forearm.	[62]	2015
	E. coli and E. faecalis (Symbio®)	Clinical trial (Children)	Subjects at severe risk of atopy	• Reduction of AD;<br>• no effect on food sensitisation was demonstrated.	An immune modulation in terms of prevention of AD in infancy was demonstrated by feeding bacterial lysates early in life.	[63]	2014

Table S1 Restoration of healthy skin microbiota (Continued. C) Microorganism-derivate substances

MICROORGANISM-DERIVATE SUBSTANCES	*S. epidermidis* ATCC12228 (cytoplasmic bacteriocin compounds)	*In vitro*	antimicrobial activity and characterisation	• selective antimicrobial activity against *S. aureus* MRSA,<br>• no active actions against S. epidermidis, E. coli, and Salmonella Typhimurium.	Promising *S. aureus* growth inhibition agent with a great potential for topical AD treatment.	[68]	2020
	L. paracasei IJH-SONE68 (esopolysaccarides)	BALB/cAJcl mice	PiCl-Induced Delayed-Type Allergy Mouse Model / Oral	• Inhibition of the catalytic activity of hyaluronidase;<br>• overexpression of ear interleukin-4 (T2 helper cytokine);<br>• increase in serum immunoglobulin E.	Reduction in the ear swelling in mice. Improvement in type I and IV allergies as well as AD.	[69]	2019
	Malassezia globosa CBS7966 (secreted protease)	*In vivo* and *in vitro* studies	Skin Sampling and Protease characterisation	• The protease is expressed on Human Facial Skin;<br>• *S. aureus* biofilm disruption by hydrolysing *S. aureus* protein A.	Definition of the role of *Malassezia* and its enzymes for human skin health.	[70].	2018
	C. granulosum (Bacterial cell wall fragment P40 conjugated with jaluronic acid)	Female mice	Oxazolone-induced contact AD / Topical	• Reduction in ear thickness and weight together with oedema;<br>• anti-inflammatory effects confirmed by histological analysis;<br>• leukocyte recruitment.	The use of this cream may potentially alleviate the symptoms of and/or treat irritant contact dermatitis,	[71]	2017
	Streptomices narvonensis subsp. Josamyceticus (bacteriocins)	NC/Nga Mice	Induction of AD-Like Skin Lesions / Topical	• Improvement in SCORAD;<br>• decrease in the density of cellular infiltration into the dermis and the serum IgE level;<br>• reduced the expression of IFN-γ and IL-4 in auricular lymph node cells and the skin lesions.	Topical application of josamycin to AD lesions colonised by *S. aureus* would be beneficial for control of AD by acting on superficially located *S. aureus* and inhibiting the development of Th1 and Th2 cells	[72]	2017

MICROORGANISM-DERIVATE SUBSTANCES	*S. lugdunensis* IVK28 (bacteriocins)	*In vitro*	Antimicrobial activity and characterisation	• novel thiazolidine-containing cyclic peptide antibiotic that prohibits colonisation by *S. aureus*;<br>• non-ribosomally synthesised bioactive compound from human-associated bacteria.	Human microbiota should be considered as a source for new antibiotics.	[73]	2016
	L. casei KCTC 12398BP (P14 protein)	NC/Nga mice	AD induced by cream prepared from house dust mites and a crude extract allergen of *D. farinae* / Topical	• Down regulation of serum IgE and interleukin-4;<br>• improvement in SCORAD and scratching score.	Potential therapeutic effects and use as immunomodulatory agent for clinical treatment.	[74]	2015

Table S1 Restoration of healthy skin microbiota (Continued. D) Microbiome transplantation

MICROBIOME TRANSPLANTATION	Skin microbiota communities	Pilot study (Adults)	Healthy volunteers / Complete transplant	• Evidence of transfer of a partial DNA signature were demonstrated.	Unenriched transfer of whole cutaneous microbiota, despite the challenges, is worthy of further investigation to restore the dysbiosis that occurs in AD	[76]	2019
	R. mucosa<br>ATCC BAA-692	Clinical trial (Adults and paediatric)	AD diagnosis /<br>Allogeneic transplant	• Improvement in SCORAD;<br>• decrease of pruritus, topical corticosteroid use and *S. aureus* colonisation;<br>• no adverse events reported.	These early results support continued evaluation of *R. mucosa* therapy with a placebo-controlled trial.	[77]	2018
	Staphylococcus hominis and *S. epidermidis*	Clinical trial (Adults)	AD diagnosis /<br>Autologous transplant	• Decreased *S. aureus* colonisation was observed at the autologous microbiome transplant site compared to the vehicle-treated contralateral forearm.	A single application was sufficient to exert antimicrobial action.	[19]	2017

Table S2 Drug Delivery System (DDS) for AD treatment. A) Nanoparticles (NPs)

	Vehicle and compound	Methodology and model	Target/Characterisation	Results	Conclusion	Ref.	Year
NANOPARTICLES	Betamethasone valerate (BMV) nanoencapsulate into the chitosan nanoparticles (CS-NPs)	*In vitro* formulation and characterisation<br>*Ex-vivo* with Wistar albino rat skin	• Drug permeation studies<br>• retention into various skin layers	• Facilitated drug penetration across the *stratum corneum*<br>• higher drug retention into various skin layers (epidermis and dermis)	Increased localised targeting and improved therapeutic efficacy for treatment of AD.	[88]	2019
	Tacrolimus-loaded thermosensitive solid lipid nanoparticles (SLN)	*In vitro*<br>formulation and characterisation<br>*Ex vivo*<br>Sprague Dawley rat dorsal skin<br>and *in vivo*<br>New Zealand white rabbits	• Skin penetration tests	• Penetration to a deeper layer than the reference product;<br>• *D*elivering more drug into deeper skin layers than the controls.	Potential application for the delivery of difficult-to-permeate, poorly water-soluble drugs into deep skin layers.	[89]	2019
	Hyaluronic acid-modified betamethasone encapsulated polymeric nanoparticles (HA-BMV-CS-NPs)	*In vitro* formulation and characterisation<br>*ex vivo* (Wistar albino rat skin)	• Physicochemical characteristics<br>• Release study<br>• Drug permeation	• *In vitro* release study displayed Fickian diffusion-type mechanism of release in simulated skin surface<br>• Higher amount of drug retained in the epidermis and the dermis compared to compound alone	Efficient dermal targeting of betamethasone and improved anti-AD efficacy.	[90]	2019

NANOPARTICLES	Hydrocortisone hydroxytyrosol anti-oxidant-loaded chitosan nanoparticles (HA-HT-CSNPs)	*In vivo*<br>Human adult healthy female	• Systemic toxicity	• Not significative differences in parameters level indicating non systemic toxicity	Safe, well-tolerated, and non-toxic, which may be useful in treating AD.	[91]	2019
	Tacrolimus nanoparticles based on chitosan combined with nicotinamide (FK506-NIC-CS-NPs)	*In vitro* formulation and characterisation<br>*In vivo* BABL/c mice<br>(induced skin lesion)	• Skin permeation studies	• Enhanced permeation through and into the skin,<br>• Efficacy on clinical symptoms	The system enhances the permeability and plays an adjuvant role in anti-AD by reducing the dose of active principle.	[92]	2018
	Betamethasone Valerate incorporate in lipid carriers	*In vitro* formulation and characterisation<br>*Ex vivo* and preclinical testing	• Permeation studies<br>• Anti-inflammatory testing	• Enhancement permeation ratio compared to plain gel.<br>• Significant extended anti-inflammatory effect	The developed formulation is efficient in a once a day dose in therapy for AD.	[93]	2018
	Hyaluronic acid (HA) decorated tacrolimus-loaded nanoparticles (TCS-CS-NPs)	*In vitro* formulation and characterisation<br>Ex vivo and *in vivo*<br>NC/Nga mice skin	• Drug permeation<br>• Evaluation of therapeutic efficacy	• Sustained release pattern<br>• Efficient dermal targeting<br>• Improved therapeutic efficacy	This formula may be a promising therapeutic approach for rationalised management of AD, particularly in children as well as in adults with steroid phobia.	[94]	2018
	Dendritic Core-Multishell Nanocarriers (CMS)	*In vitro* formulation and characterisation<br>*in vivo* SKH-1 mice<br>(AD induced by oxazolone)	• Topical Application<br>• Subcutaneous Injection	• Topical Application accumulate in the *Stratum Corneum* only<br>• Biocompatibility<br>• No evidence of toxicity	Suitable candidates for drugs encapsulation targeting stratum corneal without carrier penetration and thus without biological effects by the carrier itself	[95]	2017

**NANOPARTICLES (NPs)**	Cationic polymeric chitosan nanoparticles (CSNPs) loaded with hydrocortisone (HC) and hydroxytyrosol (HT)	*In vitro* formulation and characterisation and antimicrobial activity<br>*In vivo*<br>Albino Wistar rats	• Sub-chronic dermal toxicity	• Significant target delivery<br>• Lower systemic drug absorption than the commercial formulation<br>• Improved drug accumulation and bioavailability	Beneficial and safe for patients with AD	[96]	2016
	Nanocarrier–based transcutaneous co-delivery of hydrocortisone (HC) and hydroxytyrosol (HT)	*In vitro* formulation and characterisation<br>*In vivo*<br>NC/Nga mice<br>(AD lesion induced by 1–chloro–2, 4–dinitrobenzene) (DNCB)	• Clinical efficacy<br>• Immunological studies<br>• Histological examinations	• Improve SCORAD<br>• Decrease in IgE and PGE_2expression<br>• Reduced histamine and VEGF-α levels in serum and skin homogenates<br>• Inhibition of inflammatory cell chemotaxis and infiltration.	Alternative therapeutic approach in the management of dermatosis.	[97]	2014
	Silver-nanolipid complex (sNLC)	*In vitro* formulation, characterisation and antimicrobial activity<br>*In vivo* BALB/c mice<br>murine animal (model of AD)	• Anti-inflammatory activity evaluation	• High adhesivity to skin and bacterial surfaces,<br>• Locally high concentrations of silver ion killing the bacteria<br>• Restoration of the distorted skin barrier, much more effective than silver alone	NLC incorporation makes the drugs more effective (penetration enhancement) and simultaneously exploits the skin normalisation ability.	[16]	2014

**NANOPARTICLES**	Hydrocortisone-loaded chitosan nanoparticles	*In vitro* formulation and characterisation<br>*in vivo*<br>NC/Nga mice<br>(AD lesion induced by 1–chloro–2, 4–dinitrobenzene) (DNCB)	• Evaluation of dermatitis severity<br>• Relative expression of IgE, histamine, PGE_2 and VEGF-α<br>• Procarta® immunoassay	• Improvement in SCORAD<br>• Decrease in IgE and PGE_2expression<br>• Reduced histamine and VEGF-α levels in serum and skin homogenates<br>• Inhibition of inflammatory cell chemotaxis and infiltration at the site of inflammation.	Effective therapeutic approach to manage dermatitis.	[98]	2014

Table S2 DDS for AD treatment (Continued. B) Liposomes, ethosomes and vesicles

LIPOSOMES, ETHOSOMES AND VESICLES	Ultra-flexible lipid vesicles to deliver Cyclosporin (CyA)	*In vitro* formulation and characterisation<br>Franz diffusion cell<br>(human heat-separated epidermis)	• Absorption study	• Formulations facilitated CyA permeation through the epidermis	Topical delivery of CyA is possible using the formulations designed as an alternative to the current oral or parenteral routes	[102]	2019
	β- cycloethosomes with Fluocinolone acetonide (FA)	*In vitro* formulation and characterisation<br>*Ex vivo*<br>Albino Wistar rat skin	• Skin permeability	• Able to reach the required target flux without the help of an additional penetration enhancer	Stable and efficient vesicular carrier for topical delivery with higher entrapment efficiency and stability than reference vesicles.	[103]	2018
	Liposomal polyvinylpyrrolidone-iodine hydrogel	Clinical trial<br>(Adults)	• Several skin conditions including AD	• Well tolerated formulation and led to improvements in pain, quality of life, eczema area and severity	Potential use as an effective treatment for inflammatory skin conditions associated with bacterial colonisation.	[104]	2017
	Nanoethosomal glycolic vesicles of triamcinolone acetonide	*In vitro* formulation and characterisation<br>*Ex vivo*<br>Franz diffusion cell<br>(Wistar rat skin)	• Skin permeability	• High permeation<br>• Non-irritant potential	Stable and efficient carrier for enhanced topical delivery that exhibited higher entrapment efficiency, and stability than reference ethosomal vesicles.	[105]	2017
	Ethosomes-based topical delivery system of cetirizine	*In vitro* formulation and characterisation<br>*In vivo* and *Ex vivo*<br>BALB/c mice<br>(AD lesion induced by oxazolone)	• Skin permeation and deposition<br>• Skin sensitivity<br>• Pharmacodynamic evaluation	• Reduction in scratching score, erythema score, skin hyperplasia and dermal eosinophil count	Formulation of effective carriers for dermal delivery of antihistaminic drug,<br>cetirizine, for the treatment of AD.	[106]	2014
	Levocetirizine based on flexible vesicles<br>(FVs)	*In vitro* formulation and characterisation<br>*In vivo* and Ex vivo<br>BALB/c mice<br>(AD lesion induced by oxazolone)	• Skin permeation and deposition<br>• Pharmacodynamic evaluation	• Reduction in scratching score, erythema score, as well as dermal eosinophil count.	A novel FV based topical formulation<br>developed for treatment of AD.	[107]	2014
